# Management of Isolated Tibial Plateau Defect in the Setting of Anterior Cruciate Ligament Reconstruction and Meniscus Repair: A Case Report and Review of the Literature

**DOI:** 10.1155/cro/5136390

**Published:** 2025-06-10

**Authors:** Hillary Rolfs, Petros Frousiakis, Brent Sanderson, Brian Kwan

**Affiliations:** ^1^Community Memorial Health System, Ventura, California, USA; ^2^Community Memorial Hospital, Ventura, California, USA; ^3^University of Rochester, Victor, New York, USA

**Keywords:** cartilage defects, MACI, osteochondral defect tibial plateau

## Abstract

**Purpose:** Our work describes the use of matrix-induced autologous chondrocyte implantation (MACI) for a lateral tibial plateau osteochondral defect, in the setting of a concomitant meniscus repair and anterior cruciate ligament (ACL) reconstruction. To our knowledge, there is minimal research describing the application of MACI for cartilage defects of the tibial plateau, which brings about its own challenges.

**Methods:** A 26-year-old male presented with lateral right knee pain and instability following a soccer injury. A bucket-handle lateral meniscus tear, 2 cm by 2 cm osteochondral defect of the lateral tibial plateau, and complete ACL tear were identified on magnetic resonance imaging (MRI). Our case vignette describes a two-stage MACI procedure for an osteochondral defect of the lateral tibial plateau with concurrent all-inside meniscus repair and ACL reconstruction using bone-patellar tendon-bone (BTB) autograft.

**Results:** Follow up period was 20 months with excellent patient satisfaction and resolution of pain. Outcome measures (International Knee Documentation Committee (IKDC)) and Knee Injury and Osteoarthritis Outcome Score (KOOS) returned to preinjury levels at 8.5 months. Return to sport was achieved at 10 months postoperatively.

**Conclusion:** The miniopen MACI procedure provides a logistically reasonable technique mitigating the anatomic challenges of tibial plateau osteochondral defects and potentially providing improved long-term outcomes. It is our hope that this work will contribute to the current understanding of the treatment options for osteochondral defects of the tibial plateau.

## 1. Introduction

Matrix-induced autologous chondrocyte implantation (ACI) (MACI) is a tissue engineering technique for the treatment of osteochondral lesions. This technique has been well-described in its use for patellar and femoral chondral defects, yet tibial defects present unique geometric and anatomical challenges. There is a paucity of evidence with regard to the application of MACI for tibial defects, and microfracture remains a first-line treatment for osteochondral defects of the tibia. However, concern for the long-term outcomes following microfracture-induced fibrocartilage creation has been questioned [1]. More advanced methods such as microfracture augmentation, ACI, osteochondral allograft transplantation, or osteochondral autograft transfer (OAT) are more technically challenging but may provide improved hyaline cartilage restoration [1, 2]. In this technical note, we describe the use of MACI for a lateral tibial plateau osteochondral defect, in the setting of a concomitant meniscus repair and anterior cruciate ligament (ACL) reconstruction.

## 2. Case Report

**Figure 1:**
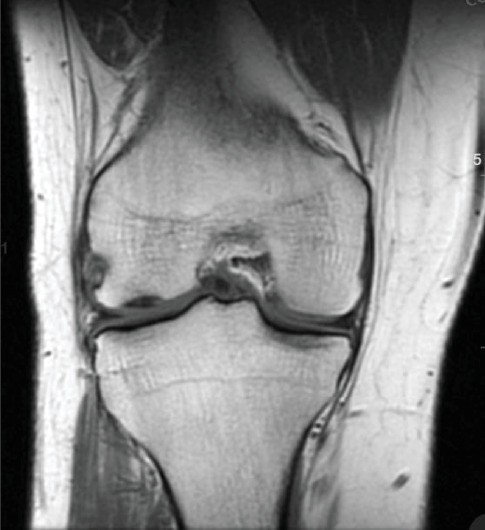
Chronic complete ACL tear, chondral injury of lateral femoral condyle.

**Figure 2:**
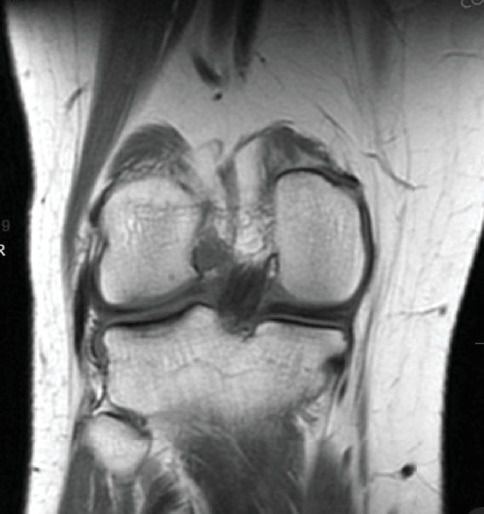
Suspected chondral injury of lateral tibial plateau.

**Figure 3:**
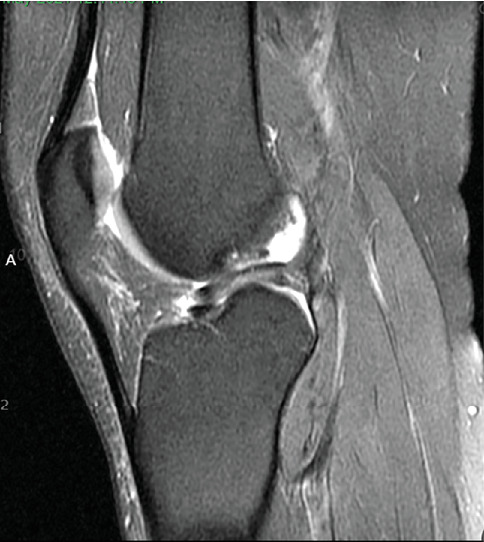
Bucket-handle tear of the lateral meniscus with a displaced fragment in the intercondylar notch.

**Figure 4:**
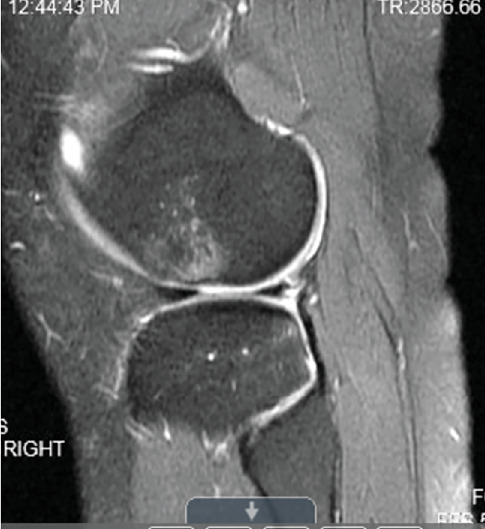
Redemonstration of lateral meniscus tear.

Patient initially presented in May of 2021 as a 26-year-old male with pain and instability of his right knee, which started after a pivoting injury that occurred while playing soccer. Patient did have a BMI of 33 but was otherwise healthy. Pertinent exam findings included no significant varus or valgus malalignment, 1+ effusion, focal tenderness to palpation of medial and lateral joint lines, negative McMurray, and grade 3B Lachman. Patient also had a negative prone dial test at 30° and 90° of flexion. Right knee radiographs demonstrated no significant varus or valgus deformity and a 7° posterior tibial slope, and no acute pathology was seen otherwise. Given normal alignment on clinical exam and radiographs, full lower extremity standing radiographs were not ordered. A bucket-handle lateral meniscus tear, osteochondral injury of the lateral femoral condyle, and complete subacute ACL tear were identified on MRI, and at that time, there was suspicion for injury to the lateral tibial plateau as well (Figures 1, 2, 3, and 4).

### 2.1. Positioning 06/15/21

Patient was brought into the operating room where he was placed supine on a flat-top table. All bony prominences were well padded, and a sequential compression device was placed on the nonoperative (left) leg. General anesthesia was induced.

### 2.2. Knee Examination Under Anesthesia 06/15/21

Clinical examination under anesthesia was performed including assessment of alignment; the patient was felt to not have any significant varus or valgus alignment. Positive 2A Lachman's and a 2+ pivot shift test were noticed. Passive range of motion was determined to be 0°–130°. The knee was placed under varus and valgus stress and felt to be stable. Posterior drawer test was performed and deemed to be negative. There were no signs of posterolateral instability.

### 2.3. Diagnostic Arthroscopy

**Figure 5:**
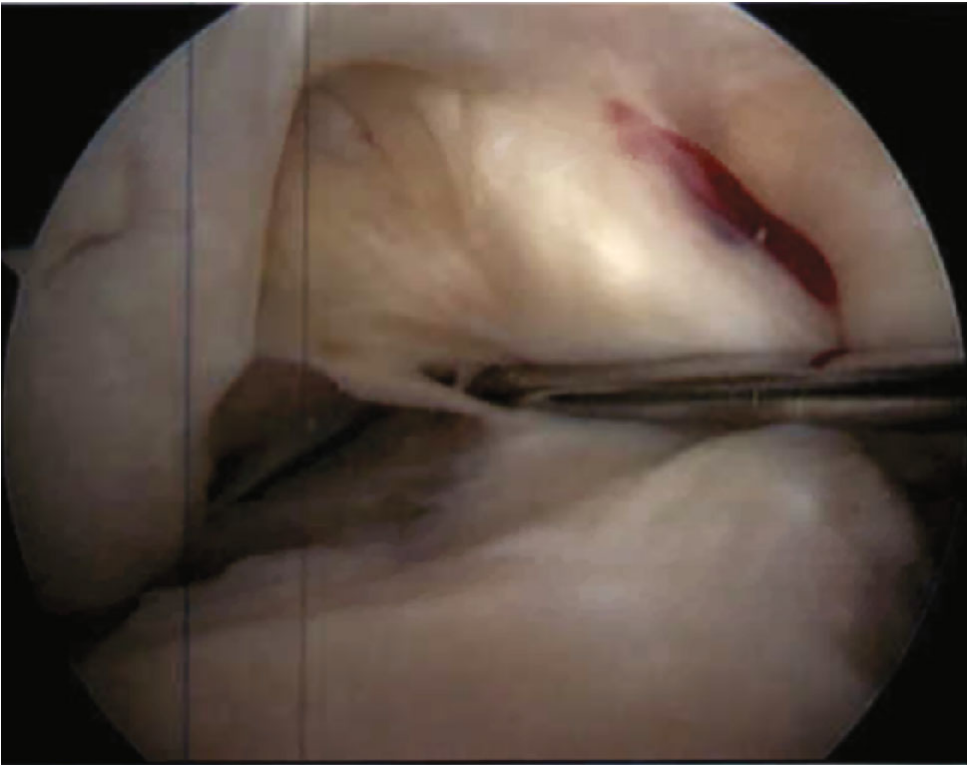
Arthroscopic image from 12/7/21 demonstrating an incompetent ACL.

**Figure 6:**
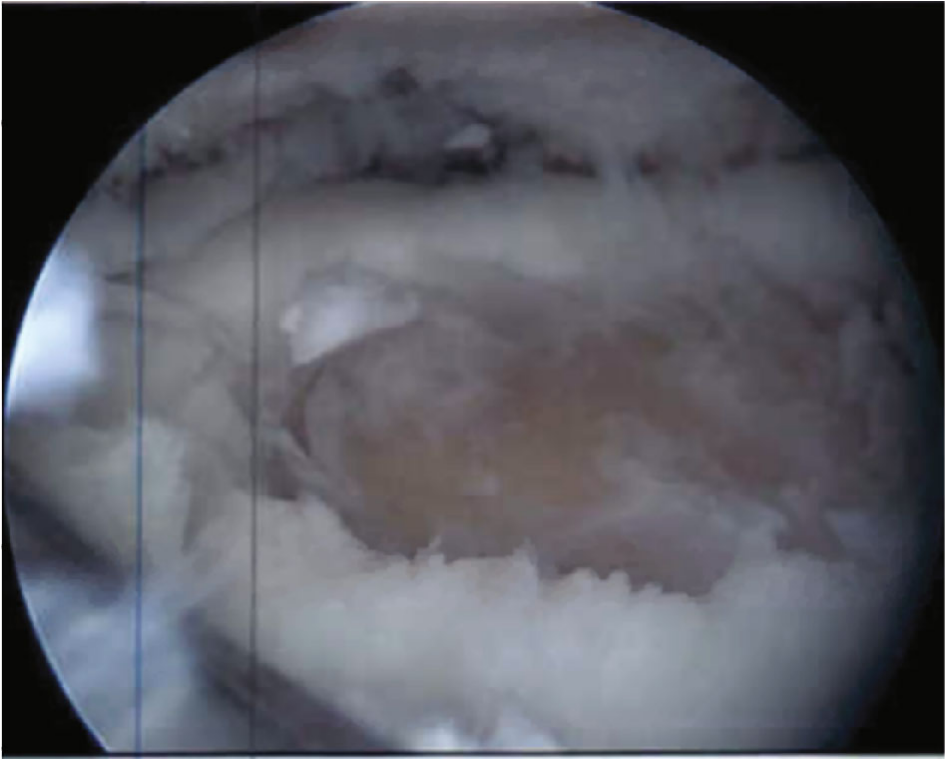
Arthroscopic image from 12/7/21 demonstrating a large cartilage defect of the lateral tibial plateau; defect has been debrided to clean edges.

Standard two-portal diagnostic arthroscopy was performed, which demonstrated no medial meniscus tear and a displaced bucket handle tear of the lateral meniscus. A full-thickness tear of the ACL was encountered (Figure 5), and the PCL was intact. A full-thickness cartilage defect (Grades III–IV) was encountered on the lateral tibial plateau; chondroplasty was performed to stabilize edges (Figure 6). There was mild cartilage softening (Grade I) on the lateral femoral condyle above the tibial plateau defect.

### 2.4. Lateral Meniscus Repair

The meniscus rasp was utilized to debride the tear and cause bleeding. The all-inside meniscus repair device was used to make two horizontal mattress stitches into the posterior body and midbody. After repair, the meniscus was stable to probing.

### 2.5. Cartilage Biopsy

Ringed curette was utilized to obtain a cartilage biopsy from the nonweight-bearing portion of the femoral notch in preparation for MACI procedure.

### 2.6. Postoperative Course

Patient was made nonweightbearing of his right lower extremity with crutches in the immediate postoperative period. He followed up in our clinic in 2 weeks following surgery. He remained nonweightbearing to the right lower extremity for 6 weeks and then was slowly advanced to be weight-bearing as tolerated over the subsequent few weeks. The patient had additional follow-up and was seen in our office for preoperative evaluation prior to undergoing the second stage of the planned two-stage procedure.

### 2.7. Positioning 12/07/21

Patient was brought into the operating room and placed supine on a flat-top table. All bony prominences were well padded, and a sequential compression device was placed on the nonoperative leg. General anesthesia was induced.

### 2.8. Knee Examination Under Anesthesia

Examination under anesthesia was performed which again demonstrated no notable malalignment in the coronal plane, a Grade 2B Lachman's and 2+ pivot shift test. Knee range of motion was determined to be 0°–140°. There was no evidence of instability to varus or valgus stress, and a negative posterior drawer exam was confirmed.

### 2.9. Patellar Tendon Autograft Harvest

Incision was marked just medial to midline overlying the patella from the inferior pole to the tibial tubercle, approximately 6 cm in length. Incision was made using a 15-blade scalpel down to the paratenon. The paratenon was incised and dissected until the patellar tendon was clearly defined. Subsequently, the central one-third of the patellar tendon was harvested with bone blocks from both the patella and tibial tubercle. The graft was prepared on the back table with a 10 mm by 22 mm bone block for the femur and a 10 mm by 25 mm bone block for the tibia. Two #2 fiberwires were placed through the bone block for the tibia, and one #2 fiberwire was placed through the bone block for the femur. The graft was then appropriately tensioned.

### 2.10. Diagnostic Arthroscopy and Partial Meniscectomy

Tourniquet was inflated and a standard two-portal diagnostic arthroscopy was performed with the portals placed within the initial BTB harvest incision. A newly encountered degenerative-appearing lateral meniscus radial tear through the posterior horn and body was identified, with prior repair remaining intact; this area was debrided to a stable base using the shaver and punches. The lateral meniscus was then further probed and found to be stable. The ACL remnant was found to be scarred down to the PCL; this was then debrided using a shaver. The ACL footprints on both the tibia and femur were then identified and marked using a cautery device. Minimal lateral notchplasty was performed using a shaver and burr.

### 2.11. ACL Reconstruction With BTB Autograft

The femoral tunnel was drilled to a depth of 25 mm using a 10 mm drill; intact posterior and lateral walls were confirmed with direct visualization. A notcher was used to notch the femoral tunnel. The tibial guide set at 60 degrees was then introduced over the previously marked anatomic ACL tibial footprint, and the tibial tunnel was drilled using a 10-mm core reamer. The back wall of the tunnel was smoothed using a rasp. The periosteum surrounding the outer cortex of the tibial tunnel was cleared to assist with graft passage. Shuttling suture was utilized to pass the graft through the tibial tunnel into the femoral tunnel; this was then fixed with a 7 mm by 20 mm metal interference screw. The knee was subsequently cycled 30 times through full range of motion to remove creep.

### 2.12. Lateral Tibial Plateau MACI Procedure

**Figure 7:**
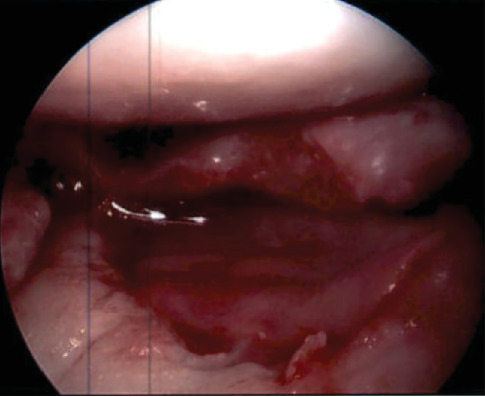
Prior lateral tibial plateau cartilage defect now filled with MACI graft held with fibrin glue.

Attention was turned to the lateral tibial plateau osteochondral defect, which was found to measure 2 cm by 2 cm and had Grades III–IV cartilage defect with exposed subchondral bone. A ringed curette and the arthroscopic mechanical shaver were used to remove damaged cartilage to the level of healthy cancellous bone, taking care to bring the borders of the defect to a 90° angle of healthy cartilage. The calcified cartilage zone was completely removed, and the defect had a stable base and edges. The defect was then measured and found to be 2 cm by 2 cm. A small 3-cm lateral parapatellar arthrotomy was made to aid in inserting the graft into the defect. The prepared defect bed was dried using thrombin-soaked pads, and fibrin glue was placed in a thin layer. This was followed by the prepared 2 cm by 2 cm MACI graft. The graft was held in place using a peanut for 3 min, and the knee was subsequently cycled approximately 20 times. The scope was reintroduced into the knee joint to evaluate the graft, which remained stable in its appropriate position (Figure 7).

### 2.13. Tibial Tunnel Interference Screw

**Figure 8:**
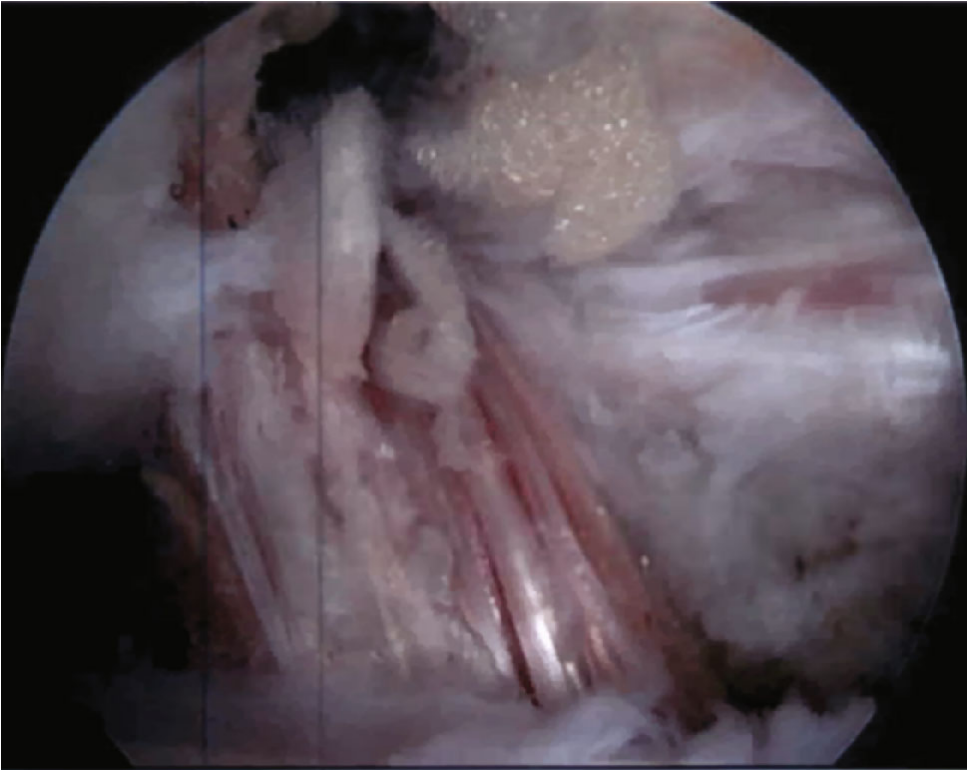
Reconstruction of the ACL with bone-patellar tendon-bone autograft.

The knee was placed in about 20 degrees of flexion and appropriate tension was applied to the ACL graft as well as posterior drawer. A 9 mm by 23 mm PEEK interference screw was placed into the tibial tunnel. The knee was again taken through full range of motion without loosening or impingement. Lachman's test and pivot shift were performed and noted to be stable. The arthroscope was reinserted, and graft appeared stable to probing with appropriate tension and location (Figure 8).

### 2.14. Rehabilitation Protocol

Following the procedure, the patient was kept nonweightbearing for a total of 4 weeks; he was subsequently progressed to weight bearing as tolerated over the following 4 weeks. Passive range of motion was initiated on postoperative Day 1 from 0° to 90°. Full return to activity was allowed 6 months postoperatively.

### 2.15. Outcomes

**Figure 9:**
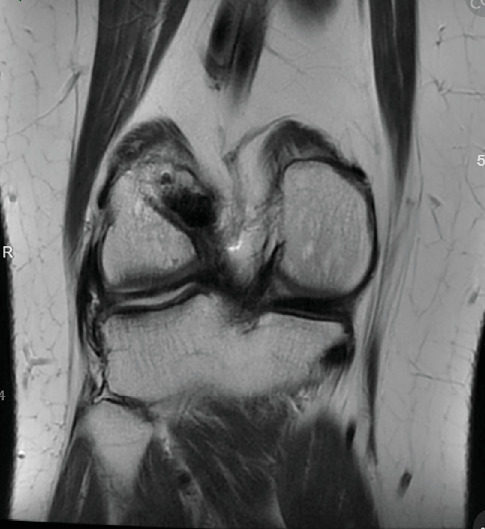
MRI from 09/2022 demonstrating fill of the lateral tibial plateau cartilage defect.

**Figure 10:**
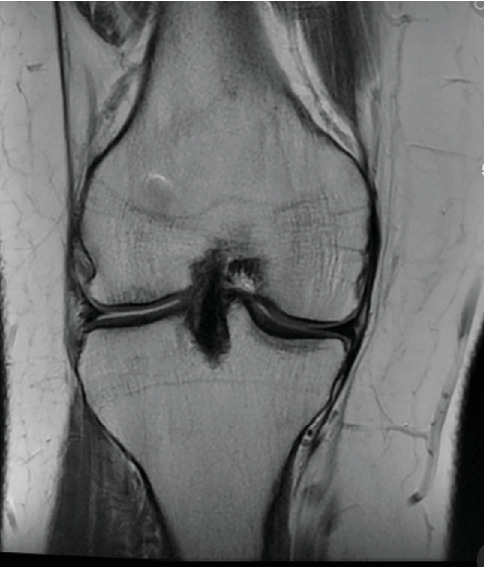
MRI from 09/2022 again demonstrating fill of the lateral tibial plateau cartilage defect.

**Figure 11:**
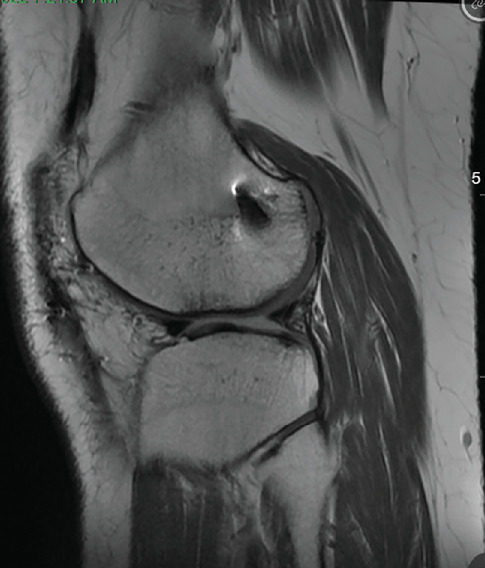
Sagittal view of MRI from 09/2022 redemonstrating fill of prior cartilage defect of lateral tibial plateau.

**Figure 12:**
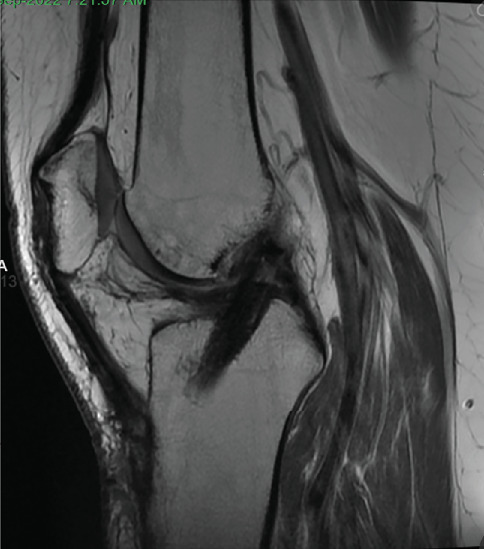
MRI from 09/2022 demonstrating intact ACL reconstruction.

Patient was most recently seen 9 months postoperatively; at that time, he noted no knee pain but did note occasional stiffness. Patient's active range of motion was 0–135 without pain. He had no joint line tenderness and negative McMurray's, as well as negative Lachman and anterior drawer testing. International Knee Documentation Committee Subjective knee form (IKDC) was 77%, and Knee Injury and Osteoarthritis Outcome Score (KOOS) was 88.7%. He had been previously progressed to full activity. MRI was obtained 09/27/2022 which demonstrated filling of cartilage defect of lateral tibial plateau and maintained ACL reconstruction (Figures 9, 10, 11, and 12).

## 3. Discussion

Focal tibial cartilage defects will typically present with localized knee joint line pain with weight-bearing and can be associated with effusions or mechanical symptoms such as locking or catching [3]. The physical exam may be inconclusive, but patients with tibial plateau cartilage defects may have focal joint line tenderness to palpation. Full-length lower extremity radiographs should be obtained, and persons with abnormal limb alignment should be considered for osteotomy. Magnetic resonance imaging (MRI) is the imaging method of choice to evaluate for osteochondral defects, cartilage lesions, and subchondral bone edema [3, 4]. However, MRI is not as sensitive as one might think; studies have shown that even chondral lesions of 50% or greater depth sensitivity range from 73 to 96% [5]. Another study showed that approximately 20% of chondral lesions have the same signal intensity as normal cartilage on MRI [6].

Tibial osteochondral defects currently present a challenge as there is no clear treatment algorithm. Multiple factors come into play when choosing a treatment, including but not limited to the age of the patient, size and location of the defect, comorbidities, activity level, BMI, other knee injuries, and alignment. For patients with defects of the medial femur or tibia and varus alignment, valgus-producing osteotomies are an established treatment that is now being utilized in combination with other procedures mentioned below [7–9]. There are six main procedures (excluding arthroplasty) performed for osteochondral defects of the knee: chondroplasty, marrow-stimulating techniques, OAT, osteochondral allograft transfer (OCA), ACI/MACI [1].

Marrow stimulation techniques, classically consisting of microfracture, are technically easy techniques that are low cost and should be considered in an active patient who is compliant with rehabilitation that has a defect < 2 cm without subchondral plate involvement [1, 10–13]. The downside to microfracture is the predominant production of type I fibrocartilage instead of Type II hyaline cartilage, which brings into question the long-term efficacy of this technique [10–14]. Cellular augments are also being studied in combination with microfracture techniques, including bone marrow aspiration (BMC) and mobilized peripheral blood stem cells; this has shown early promise as a treatment strategy [15, 16] .

OAT is a technique where graft is harvested from a nonweight bearing portion of the patient's knee and implanted into the defect. Mosaicplasty is similar in that it harvests a patient's own cartilage from a nonweight bearing portion, but it harvests smaller cartilage plugs. These techniques have shown promising results for young and active patients with osteochondral defects < 3 cm, and studies have indicated a long-term benefit at > 10 years. Benefits to the OAT procedure include the shorter recovery time, quicker graft integration, and potential early return to full activity at 3 months [17]. OAT has also been found to have a high percentage of hyaline-like cartilage at the integration site when compared to other techniques [18]. However, this technique is more technically challenging with symptomatic donor site reported in 0%–92% of cases, which is a wide range [19]. A systematic review by Andrade et al. found donor site morbidity, including patellofemoral disturbances, crepitation, and effusion, occurred in 5.9% of knee-to-knee transfers with mosaicplasty [20].

OCA describes a procedure in which allograft is taken and implanted into an osteochondral defect. One benefit to this technique is it can be used for larger lesions (>2 cm) located anywhere in the knee, and more recent research has indicated long-term benefit to this procedure, with a survival rate of 78.7% at 10 years [21]. Limiting factors for this technique include cost, donor availability, technical difficulty, and an additional risk of disease transmission with using allograft tissue. The recovery from this procedure is typically drawn out to 1 year prior to return to sport, which may make it a less attractive option for certain patients.

ACI and MACI are two-stage techniques that require a biopsy of articular cartilage, which is then grown ex vivo and reimplanted into the defect. The standard MACI technique involves growing cultures of the chondrocyte biopsies for 3–4 weeks in a Type I/III collagen scaffold matrix. The lab grown cartilage is then implanted into the osteochondral defect and held by a fibrin glue. MACI has been shown to have good outcomes with improved patient-reported outcomes and improved survivorship, but long-term data continues to be lacking [22]. MACI has traditionally been thought to be a more expensive procedure, with one study finding the total average cost to be €23,450 compared to €14,563 for patients treated with microfracture [17]. However, there has been a question of total cost effectiveness, which was examined in a study by Vogelmann et al. out of Germany that demonstrated improved long-term outcomes compared to other interventions (microfracture, matrix-associated bone marrow stimulation, and partial knee replacement) [23]. They reported that 82% of patients undergoing MACI required additional surgery at an average of 17 years after MACI, and 5.5% of these patients went on to have total knee arthroplasty (TKA). This is compared to patients undergoing the interventions mentioned above, where 86% of patients underwent additional procedures at an average of 12 years after the index procedure, and 26% of patients underwent TKA. Again, patient-specific factors should be considered as this is a costly and invasive procedure that requires a two-stage procedure [23].

In our patient's case, after thorough review of imaging and discussion with the patient, it was believed that MACI was the indicated procedure for his injury. Additionally, it should be noted that MACI performed at the time of ACL reconstruction has been reported to have satisfactory patient reported outcomes and function [24].

As more techniques are developed and more data is published on current techniques, there will be continued innovation and improved long-term patient outcomes. Individualized patient care is critical when deciding the proper technique to utilize for osteochondral defects of the tibia. Overall, the presented technique provides an argument in favor of a technically feasible two-stage MACI implantation for osteochondral defect of the lateral tibial plateau in the setting of a concomitant ACL reconstruction.

### 3.1. Advantages and Disadvantages of Surgical Technique

Advantages:

‐ MACI has been found to be the gold standard treatment for osteochondral defects > 2 cm
‐ Reconstruction of the osteochondral unit using MACI scaffold
‐ Ability to perform MACI arthroscopically; application of technique in lesions that were previously difficult to access

Disadvantages:

‐ May be cost prohibiting for some patients
‐ Technically challenging procedure
‐ Slower return to sport and activity due to the need for MACI to integrate two-stage procedure

### 3.2. Pearls and Pitfalls of Surgical Technique

Pearls:

‐ Debride osteochondral defect to establish definitive and stable borders
‐ Place thrombin soaked pads within debrided osteochondral defect in order to dry out area to prepare for MACI graft
‐ Utilize fibrin glue to cover MACI graft and to help secure the graft in place.
‐ Hold MACI graft using a peanut for a minimum of 3 min to ensure successful installment
‐ Perform MACI prior to performing ACL reconstruction to have access to the tibial plateau without damaging ACL reconstruction

Pitfalls

‐ Size MACI graft appropriately; avoid overfill or underfill of defect
‐ Initial biopsy for MACI should be of non-weightbearing portions of the femur; even so, this can lead to donor site morbidity.
‐ If there is subchondral bone involvement, OATs may be the more appropriate procedure

## Data Availability

Data sharing is not applicable to this article, as no datasets were generated or analyzed during the current study.
